# Role of Lactium™ in Psychodermatology: The CERTAIN Trial^#^ on Patients with Acne Vulgaris

**DOI:** 10.1155/2022/2916317

**Published:** 2022-05-04

**Authors:** Amit Shivaji Kerure, Satish Udare, Chetan Vispute

**Affiliations:** ^1^Dr. Amit Kerure Skin Clinic, Navi Mumbai 400703, India; ^2^Sparkle Skin and Aesthetic Centre, 26-A, Shanti Centre, Second Floor, Above Parichay Hotel, Sector 17, Vashi, Navi Mumbai 400703, India; ^3^Soumanasya Psychiatry Clinic, Seba CHS, Plot 5a Sector 16A, Swami Samarth Marg, Vashi, Navi Mumbai, Maharashtra 400703, India

## Abstract

Stress plays an important role in the causation and aggravation of psychodermatological conditions such as acne vulgaris. Alpha casein hydrolysate (*α*s1-casein hydrolysate; Lactium) has been shown to decrease serum cortisol levels, reduce stress-related symptoms, and promote relaxation. “This study aimed to compare the efficacy and safety of Lactium™ plus standard care to those of standard of care alone in reducing stress levels and acne severity in patients with acne vulgaris.” The C.E.R.T.A.I.N trial (Name registered with Clinical Trials Registry-India-No. CTRI/2019/01/017172) is a randomized, controlled, multicenter, open-label, two-arm, investigator-initiated clinical trial. A total of 100 patients with moderate-to-severe acne vulgaris were enrolled and randomly assigned to one of the two groups: Lactium™ plus standard care or standard care alone. Stress levels were assessed using serum cortisol levels, Investigator's Global Assessment (IGA) acne severity scale scores, Perceived Stress Scale (PSS) scores, and the Hamilton Anxiety Rating Scale (HAM-A) scores. The Dermatology Life Quality Index (DLQI) was also used to assess the impact of the skin disease on patients' quality of life. At 12 weeks, stress levels were significantly lower in group A (Nixiyax plus standard of care) than that in group B(only standard care), as measured by the change in serum cortisol levels (4.75 ± 4.46 vs. −0.24 ± 5.22). Furthermore, the mean change in PSS scores (3.09 ± 2.04 vs. 0.90 ± 2.76) and HAM-A scores (5.11 ± 1.94 vs. 1.25 ± 3.13) was significant. Patients in both arms had a significant decrease in total, inflammatory, and noninflammatory acne lesions, as well as a significant improvement in DLQI and IGA scores. In patients with moderate-to-severe acne vulgaris, Lactium™ was found to be both safe and well-tolerated. Lactium™ plus standard care is more effective than standard care alone in reducing acne severity through stress reduction.

## 1. Introduction

Psychosomatic disorders are physical manifestations of stress or emotional factors that can cause a disease or affect its clinical course [1]. The physical manifestations of skin disorders are the focus of psychodermatology [2, 3]. Despite its infancy, this dermatology subspecialty has piqued the interest of dermatologists and researchers. Stress is a known trigger for a variety of psychodermatological conditions, including acne vulgaris [4–7]. These conditions, in turn, may cause secondary psychiatric conditions such as emotional stress, anxiety, and depression [8], worsening the dermatological conditions and creating an unending vicious cycle [9]. Therefore, using stress reduction strategies for symptomatic relief, such as psychotherapy, cognitive behavioral therapy, relaxation techniques, music or exercise therapy, biofeedback, and hypnosis, is only logical in these conditions [2, 3]. Stress is a precipitating and aggravating factor for acne lesions [10–13], in addition to hot weather, excessive sweat, poor hygiene, smoking, alcohol intake, or chocolate [14–17]. The underlying mechanisms include overexpression of the corticotropin-releasing hormone system, activation of inflammatory and immunological processes, and neuropeptide action [4].

Acne, particularly in the young population, can cause severe distress, resulting in poor self-image, depression, and anxiety, as well as uncertainty [18–20], and has a negative impact on quality of life (QOL) [21–23]. Acne's consequences worsen its severity and frequency. The central tenets of acne treatment include benzoyl peroxide, topical or oral retinoids such as isotretinoin, antibiotics, and oral spironolactone [24]. A majority of these are associated with adverse effects and are especially dangerous during pregnancy and lactation [25]. Furthermore, they do not address the emotional aspects of psychodermatological conditions.

Because of the role of stress in the development and aggravation of acne vulgaris, stress-relieving strategies may be used as a treatment option. Pharmacotherapies to reduce stress, anxiety, and depression include selective serotonin reuptake inhibitors and other antidepressants, benzodiazepines, propranolol, morphine, hydrocortisone, and docosahexaenoic acid, which, while effective in some patients, have serious adverse effects [26]. To break the vicious cycle of stress-acne-stress, a safe and effective treatment modality is required to relieve stress in patients with acne.

Several studies have evaluated the effects of nutrient supplements on stress reduction [27–31]. Our investigational product, Lactium (*α*s1-casein hydrolysate) is a milk protein. Lactium™ is a well-researched biomolecule with anxiolytic-like properties. It is a clinically proven antistress therapy that has been available in over 120 countries for over 10 years and is patented in Europe, the United States, and Japan. Lactium™ binds to gamma aminobutyric acid (GABA-A)-A receptors in the central nervous system. The GABA-A receptor, which comprises at least 19 different subunits [32], has three binding sites, namely, *ω*1, *ω*2, and *ω*3. Lactium selectively binds to the *ω*2 binding site, increasing membrane chloride conductance, causing an influx of Cl^−^ and membrane hyperpolarization. This results in a decrease in neurotransmission and the regulation of anxiety and stress without sedative effects [33].

Several preclinical [34–36] and clinical studies [27, 31, 37–39] have found that Lactium™ lowers serum cortisol levels, improves sleep quality and efficiency, and reduces anxiety, general fatigue, and stress symptoms related to digestion and intellectual, emotional, and social problems. A separate study on its efficacy against chronic stress, conducted at Necker-Enfants Malades Hospital and BIOFORTIS, confirmed the efficacy of Lactium™ in acute stress management at higher doses (200 mg and 300 mg, respectively). As a result, Lactium™ can be used to manage certain stressful life events, such as school exams or special events in one's personal or professional life [37, 39, 40].

Lactium is a food supplement derived from milk protein (*α*s1-casein hydrolysate). We aimed to determine the efficacy of Lactium plus standard of care in comparison to that of the standard of care alone in reducing stress levels and acne severity in patients with acne vulgaris in the C.E.R.T.A.I.N trial, which is a randomized, controlled, open-label, investigator-initiated clinical study, evaluating the effects of Lactium™ plus standard of care treatment in comparison with the standard of care alone on the mental wellbeing of subjects with acne vulgaris.

## 2. Materials and Methods

### 2.1. Study Methodology

This was an investigator-initiated, randomized, controlled, multicenter, open-label, two-arm clinical trial (CTRI/2019/01/017172). It was conducted over 10 months, from January 2019 to October 2020, at Dr. Amit Kerure Skin Clinic, Navi Mumbai, and Sparkle Skin and Aesthetic Centre, Navi Mumbai. An ethics committee clearance was obtained before the commencement of the study. This study was performed in accordance with the ICH-GCP (International Council for Harmonization of Technical Requirements for Pharmaceuticals for Human Use-Good clinical practice) protocol and the applicable regulatory requirements. Written informed consent was obtained from all the participants. According to the protocol, patients with moderate-to-severe acne who met all the inclusion criteria were enrolled in the study. The patients were randomly assigned (50 : 50) to either the Lactium plus standard of care or standard of care alone groups. For all patients in both groups, the standard of care was oral doxycycline combined with topical adapalene and clindamycin gel. The primary objective of the study was to compare the efficacy of Lactium™ plus standard of care to that of the standard care alone in reducing psychological stress. The secondary objectives of the study were to compare the efficacy of Lactium™ plus standard of care to that of the standard of care alone in the improvement in the number and severity of acne vulgaris lesions using the Dermatology Life Quality Index (DLQI), as well as assess Lactium's safety and tolerability.

The primary endpoints of this study were the mean change in serum cortisol levels and the difference in stress assessment questionnaire scores between the two arms from the beginning to the end of the study. The secondary endpoints were the absolute change in lesion counts and the mean percentage change in the total, inflammatory, and noninflammatory acne lesion counts from baseline to the end of the study, as well as the change in the Investigator's Global Assessment (IGA) acne severity scale scores and DLQI scores. The safety endpoints in both arms were described as changes in laboratory parameter scores and the incidence of adverse events.

### 2.2. Inclusion and Exclusion Criteria

The study included patients over 18 years of age who were in generally good health and had a definite clinical diagnosis of moderate-to-severe acne vulgaris of grade 2, 3, or 4 on the IGA acne severity scale (see Appendix A for details on inclusion criteria). Among the exclusion criteria were known conditions that would interfere with the evaluation of acne vulgaris (see Appendix A for details on exclusion criteria).

### 2.3. Patient Randomization

A power analysis was used to calculate the sample size. One hundred patients were recruited based on the eligibility criteria. Based on the schedule generated and assigned codes, computer-generated randomization was used to assign patients to receive Lactium plus standard of care or standard of care alone. All patients in both groups received the same standard of care: oral doxycycline with topical adapalene and clindamycin gel. Because the trial was designed as an open-label study, both the doctor and the subjects knew the test product.

### 2.4. Treatment Received

Lactium™ was administered in 150 mg doses. The patients were instructed to take the prescribed drug capsule after dinner for 84 days. The patients were given a study diary in which they were asked to record the date, time, and amount of each medication taken, as well as any other medication taken and any changes in their health status.

### 2.5. Assessments and Questionnaires

Fasting blood samples were taken to determine the serum cortisol levels. The IGA acne severity scale was used to assess acne severity. The Perceived Stress Scale (PSS) and the Hamilton Anxiety Rating Scale (HAM-A) were used to assess stress, and the DLQI was used to assess the impact of skin disease on the patients' QOL. After the baseline visit (visit 1), the patients were followed up at weeks 6 (visit 2) and 12 (visit 3).

### 2.6. Statistical Analysis

All continuous study assessments were summarized according to treatment and time points using descriptive statistics (*n*, mean, median, standard deviation, minimum, and maximum). The values were considered statistically significant at *p* < 0.05. IBM SPSS version 25 (IBM Corp., Armonk, NY, USA) was used for statistical analyses.

## 3. Results

### 3.1. Patient Demographics and Baseline Characteristics

**Figure 1:**
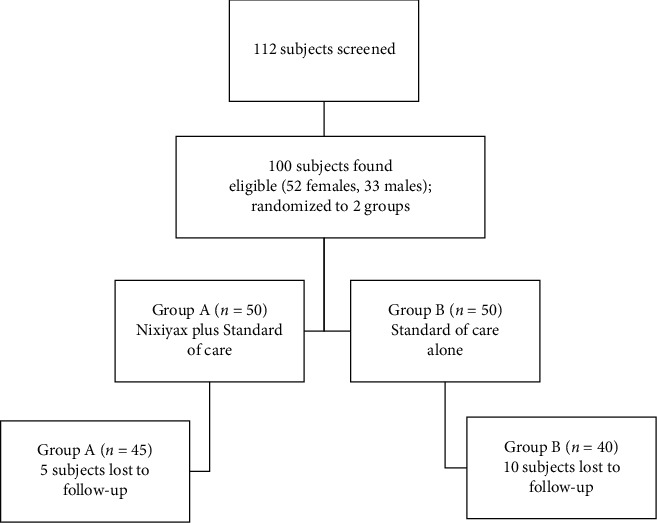
Subject recruitment and randomization.

Out of 112 patients screened, 100 were eligible for the study and were randomly assigned to either group A (*n* = 50; Lactium™ plus standard of care) or group B (*n* = 50; standard of care alone). However, only 85 patients completed the study and were included in the efficacy analysis; five patients in group A and ten patients in group B were lost to follow-up (Figure 1). A total of 52 women and 33 men participated in this study.

The mean age of the patients was 22.20 ± 3.29 years. At baseline, the demographic differences between the two groups were not statistically significant (Table 1). Serum cortisol levels were measured at weeks 6 and 12. Simultaneously, the responses to the PSS, IGA acne severity scale, HAM-A, and DLQI questionnaires and an estimate of total, inflammatory, and noninflammatory acne lesion counts were also noted.

### 3.2. Primary Endpoint Analysis

#### 3.2.1. Serum Cortisol Levels

**Figure 2:**
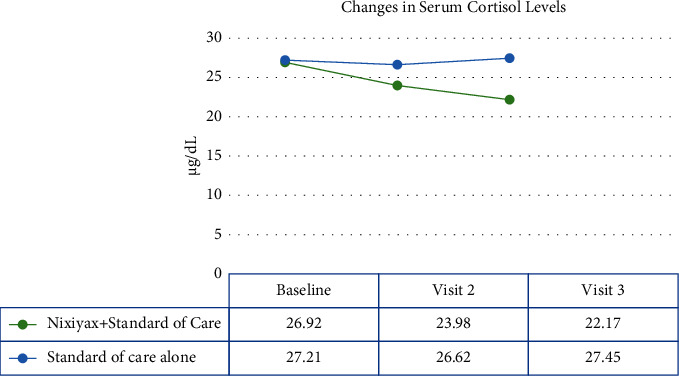
Average serum cortisol levels at baseline, visit 2, and visit 3. The change from baseline to visit 3 was significant in the Lactium plus standard of care group but not in the standard of care alone group.

**Figure 3:**
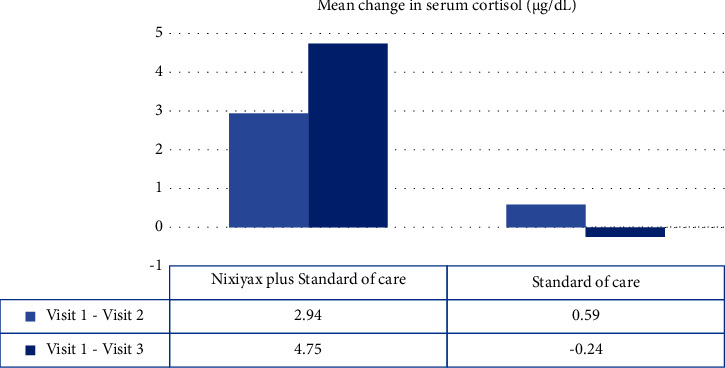
Mean change in serum cortisol (*μ*g/dL) from baseline to visits 2 and 3 in the standard of care alone group.

Serum cortisol levels in group A decreased significantly (*p* < 0.001) from baseline to visits 2 and 3. This trend, however, was not observed in group B (*p*=0.2723 and *p*=0.7750, respectively; Figure 2). The mean change in serum cortisol levels from baseline to visits 2 and 3 was significant in group A but not in group B (Figure 3).

#### 3.2.2. PSS

**Figure 4:**
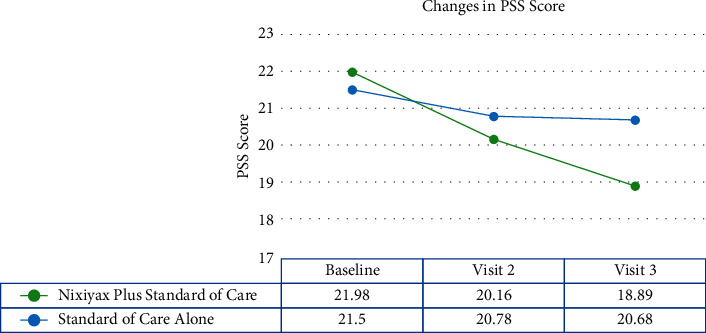
Changes in PSS scores over the course of the visits. A significant decrease in PSS scores was observed in the Lactium™ plus standard of care group, whereas the change in the PSS scores in the standard of care alone group was not significant.

**Figure 5:**
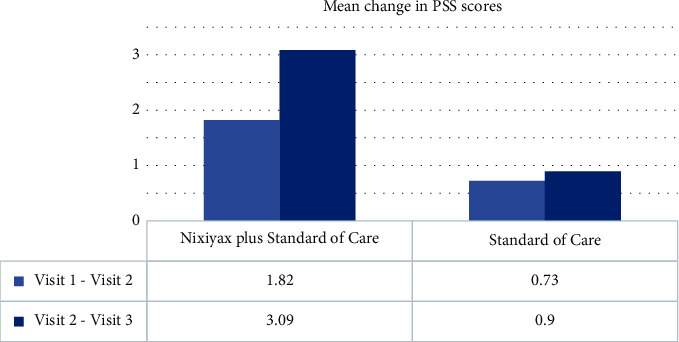
Mean change in PSS scores from baseline to visits 2 and 3.

PSS scores in group A patients decreased significantly (*p* < 0.001) from baseline to visits 2 and 3, indicating lower stress levels among the patients. In group B, however, there was a negligible decrease in PSS scores (*p*=0.0107 and *p*=0.0461, respectively; Figure 4). The mean change in PSS scores from baseline to visits 2 and 3 was significant in group A, but there was no such significant difference in group B (Figure 5). At all visits, the PSS scores were comparable between the groups.

#### 3.2.3. HAM-A

**Figure 6:**
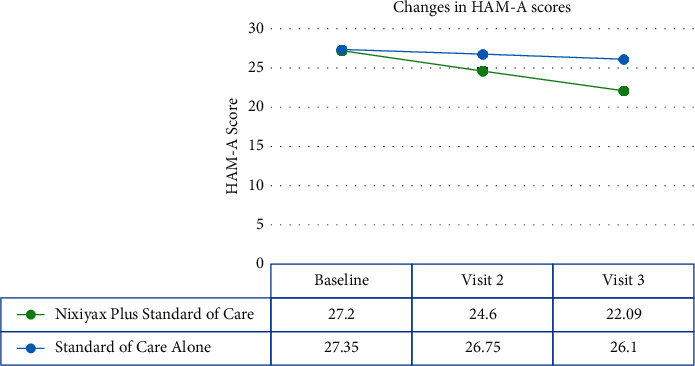
Changes in HAM-A scores. The Lactium™ plus standard of care group had lower HAM-A scores than the standard of care alone group.

The HAM-A scores in group A decreased significantly from baseline to visits 2 and 3 (*p* < 0.001), with a mean of 2.60 ± 1.74 and 5.11 ± 1.94, respectively (Figure 6). However, no statistical difference in group B's HAM-A scores was observed. The mean changes in HAM-A scores at visits 2 (0.60 ± 2.26; *p*=0.1015) and 3 (1.25 ± 3.13; *p*=0.0156), respectively, were not significant.

### 3.3. Secondary Endpoint Analysis

#### 3.3.1. Total Acne Lesion Count

**Figure 7:**
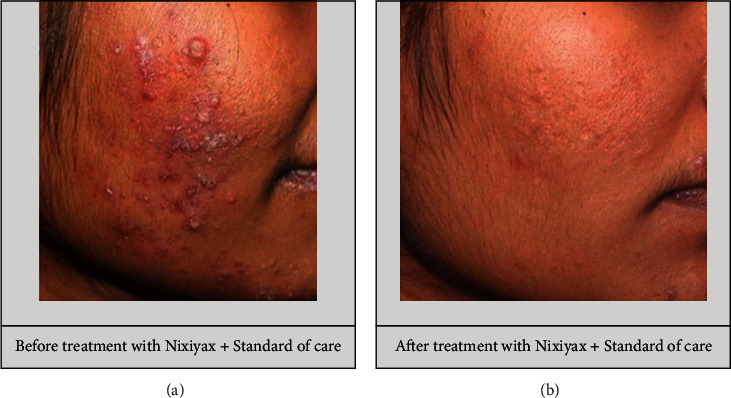
Treatment with Lactium™ plus standard of care resulted in a decrease in inflammation, the number of acne lesions, and acne severity.

**Figure 8:**
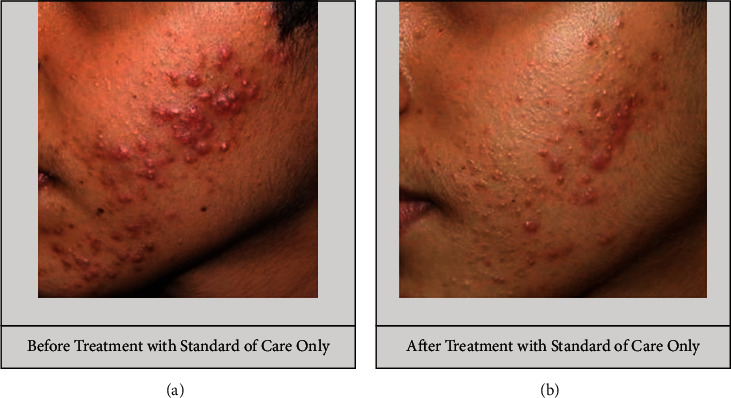
Treatment with standard of care alone did not result in a significant reduction in the number of acne lesions or acne severity.

The total acne lesion count in group A decreased significantly from baseline to visit 2, and the count further decreased at visit 3. A significant reduction in the total acne lesion count was also observed in group B over a 12 week period (Table 2). However, no significant difference in the total number of acne lesions was found between the groups (Figures 7 and 8). A nonsignificant percentage change in the total acne lesions count was observed between the groups (Table 3).

#### 3.3.2. Inflammatory Acne Lesion Count

The inflammatory acne lesions of grades 3 and 4 count decreased from baseline to visits 2 and 3 in group A. A similar trend was observed in group B (Table 2). However, there was no significant difference in the lesion count between the two groups (Figures 7 and 8). At visits 2 and 3, the percentage change in the inflammatory acne lesion count was comparable between the groups (Table 3).

#### 3.3.3. Noninflammatory Acne Lesion Count

Group A had a significant decrease in the grade 2 noninflammatory acne count from the start to the end of the study. In group B, the noninflammatory lesion count decreased significantly from baseline to the last follow-up visit (*p* < 0.001; Table 2). However, the count did not differ significantly between the groups (Figures 7 and 8). At visits 2 and 3, the percentage change in the noninflammatory acne lesion count was not significant between the two groups (Table 3).

#### 3.3.4. IGA Acne Severity Scale

From baseline to visit 3, both groups showed a significant improvement in global acne severity, as evaluated using the IGA scale, (group A: 2.67 ± 0.67 at baseline to 0.64 ± 0.57 at visit 3 (*p* < 0.001); group B: 2.73 ± 0.64 at baseline to 0.75 ± 0.54 at visit 3 (*p* < 0.001)) (Figures 7 and 8).

#### 3.3.5. DLQI

Both groups showed a significant improvement in their DLQI scores from baseline to visit 3 (group A: 13.73 ± 2.07 at baseline to 10.73 ± 1.99 at visit 3 (*p* < 0.001); 13.78 ± 1.85 at baseline to 11.15 ± 1.93 at visit 3 (*p* < 0.001)). However, the difference between the groups was not statistically significant.

### 3.4. Safety Results

There was no clinically significant change in the physical examination findings observed during the study. In groups A and B, the vital signs, clinical chemistry, and complete blood count parameters were all within the normal range.

A total of 21 adverse events were reported by 11 patients; six patients in group A reported 12 adverse events, while five patients in group B reported 9 adverse events (Table 4). The most common adverse events were fever, headache, body ache, and gastritis, all of which were managed well. The reported adverse events were mild events with no severe adverse events.

## 4. Discussion

This study is the first to show that Lactium can reduce acne severity by lowering stress levels in acne patients. According to the findings of this study, Lactium combined with the standard of care was more effective than the standard of care alone in patients with moderate-to-severe acne vulgaris. Lactium improved dermatology-related QOL while remaining safe and well-tolerated. These findings reiterate the stress-reduction findings from PROCLAIM [39], CRSSA [38], and BIOFORTIS studies [40]. The findings could be useful in the treatment of psychodermatological conditions.

Our study found a decrease in serum cortisol levels and a corresponding decrease in acne severity, highlighting the well-documented causal relationship between stress and acne [12, 13, 37, 41]. This finding also supports the notion that stress-reduction strategies can be an important component in managing psychodermatological conditions. The decrease in HAM-A scores in the investigational group in our study demonstrated the anxiolytic-like effect of Lactium; a similar decrease in anxiety scores was noted in other studies [39, 42, 43]. These findings support the beneficial role of Lactium in treating a wide range of psychodermatological conditions in which anxiety is a common factor.

Some studies have linked stress reduction techniques to a reduction in acne severity [12, 44, 45]; our findings support this theory. The PSS, HAM, and DLQI are some of the tools used to assess the impact of treatment and compare it to the baseline [46–50]. Many of the questions in these tools reflect the patients' perceptions about uncertainty and their lack of control over their symptoms and related QOL, which is commonly associated with acne-related stress and anxiety [18, 19]. The decrease in patients' scores on these instruments indicates an improvement in their sense of certainty regarding their condition, an increase in their perception of being in control of their lives, and an improvement in dermatological QOL.

There were no severe adverse events reported in the Lactium group. The 12 reported adverse events were mild and manageable, demonstrating Lactium's strong safety profile compared to pharmacological agents used in stress relief and acne treatment [24–26]. Lactium may be useful in the management of psychodermatological diseases. Stress reduction may positively impact the severity, duration, and clinical course of these conditions, which are otherwise difficult to manage due to recurrent episodes of flare and remission. Lactium, due to its safety profile, may be considered for relieving anxiety and stress associated with various life activities such as exams, interviews, marriage or death, and job loss, all of which tend to trigger and aggravate the psychodermatological conditions.

Our study did not include pregnant or lactating women, which could be a study limitation because Lactium's safety in this special population may need to be established. More research is needed to determine the beneficial effects of Lactium™ on other systemic conditions such as diabetes mellitus, hypertension, and coronary artery disease, all of which have stress as a risk factor.

## 5. Conclusions

Lactium™ combined with standard of care effectively reduced the severity of acne and the acne lesion count through stress reduction, thereby improving the dermatology-related QOL acne vulgaris patients. Lactium, as a nutraceutical, can be an alternative treatment for acne vulgaris. Its stress-relieving properties make it an appealing alternative for many psychodermatological conditions with stress as an etiological factor.

## Figures and Tables

**Table 1:** Baseline characteristics.

	Group A (Nixiyax plus standard of care) (*N* = 45)	Group B (standard of care) (*N* = 40)	*p*-value
Mean age (in years)	22.56 ± 3.19	21.80 ± 3.40	0.2958
Sex, *n* (%)			
Female	28 (0.33%)	24 (0.19%)	0.8338
Male	17 (0.20%)	16 (0.28%)	
Serum cortisol levels (*μ*g/dL)	26.92 ± 5.85	27.21 ± 5.08	0.8090
PSS score	21.98 ± 2.66	21.50 ± 2.52	0.3978
HAM-A score	27.20 ± 3.32	27.35 ± 3.08	0.8294
Total acne lesion count	20.9 ± 6.27	22.6 ± 6.28	0.2125
Inflammatory acne lesion count	5.96 ± 2.5	6.95 ± 3.07	0.1082
Noninflammatory acne lesion count	14.98 ± 4.97	15.73 ± 4.49	0.4688
IGA acne severity scale score	2.67 ± 0.67	2.73 ± 0.64	0.6836
DLQI score	13.73 ± 2.07	13.78 ± 1.85	0.9221

*N*, number of patients; PSS, Perceived Stress Scale; HAM-A, Hamilton Anxiety Rating Scale; IGA, Investigator's Global Assessment; DLQI, Dermatology Life Quality Index. Pearson's chi-square test was used to determine the relationship between sex and groups. The *p*-value was calculated using an independent *t*-test.

**Table 2:** Total acne lesion count during visits in both groups.

Changes in acne lesion counts	Group A	Group B
Total acne lesion count		
Baseline (visit 1)	20.9 ± 6.27	22.6 ± 6.28
Visit 2	9.78 ± 6.37	12.75 ± 5.71
Visit 3	5.07 ± 4.26	6.47 ± 4.43
Change from visit 1 to visit 2	11.13 ± 5.88 (*p* < 0.001)	9.88 ± 4.33 (*p* < 0.001)
Change from visit 1 to visit 3	15.8 ± 6.43 (*p* < 0.001)	16.1 ± 4.9 (*p* < 0.001)
Inflammatory acne lesion count		
Baseline (visit 1)	5.96 ± 2.5	6.95 ± 3.07
Visit 2	1.56 ± 1.77	2.48 ± 2.61
Visit 3	0.45 ± 0.84	0.8 ± 1.81
Change from visit 1 to visit 2	4.4 ± 1.81 (*p* < 0.001)	4.47 ± 2.11 (*p* < 0.001)
Change from visit 1 to visit 3	5.51 ± 2.46 (*p* < 0.001)	6.15 ± 3.42 (*p* < 0.001)
Noninflammatory acne lesion count		
Baseline (visit 1)	14.98 ± 4.97	15.73 ± 4.49
Visit 2	8.22 ± 5.26	10.28 ± 4.43
Visit 3	4.47 ± 3.79	5.38 ± 4.15
Change from visit 1 to visit 2	6.76 ± 5.49 (*p* < 0.001)	5.45 ± 3.33 (*p* < 0.001)
Change from visit 1 to visit 3	10.5 ± 5.62 (*p* < 0.001)	10.3 ± 4.56 (*p* < 0.001)

The *p*-value was calculated using an independent *t*-test.

**Table 3:** Percentage change in the total, inflammatory, and noninflammatory acne lesion counts.

	Group A	Group B	*p*-value
% Change in the total acne lesion count			
Visit 1 to visit 2	54.2 ± 26.6	44.5 ± 20.3	0.0618
Visit 1 to visit 3	75.7 ± 20.5	72.8 ± 17.7	0.4724

% Change in the inflammatory acne lesion count			
Visit 1 to visit 2	76.6 ± 21.7	66.9 ± 30.2	0.0972
Visit 1 to visit 3	91.7 ± 18.1	88.2 ± 30	0.5165

% Change in the noninflammatory acne lesion count			
Visit 1 to visit 2	44.5 ± 33.2	35 ± 23.7	0.1300
Visit 1 to visit 3	69.8 ± 25.2	66.4 ± 25.2	0.5288

The *p*-value was calculated using an independent *t*-test.

**Table 4:** Safety data.

Adverse events	Group A (Nixiyax plus standard of care) (*N* = 45)	Group B (standard of care) (*N* = 40)	Overall (*N* = 85)
Total number of AEs reported	12 (26.67)	09 (22.5)	21 (24.70)
Subjects reporting at least one AE	6 (13.33)	5 (12.5)	11 (12.94)
Total number of SAEs reported	0	0	0
Number of deaths	0	0	0

AEs, adverse events; SAEs, severe adverse events.

## Data Availability

The data that support the findings of this study are available and will be provided upon reasonable request.

## Appendix

### A. Diagnosis and main criteria for inclusion and exclusion

Inclusion
  1. Male or female patients over the age of 18 who are in generally good health.
  2. Definite clinical diagnosis of mild to severe acne vulgaris (Grade 2, Grade 3, or Grade 4 on the Investigator's Global Assessment (IGA) of acne severity.
  3. Willing and able to give informed consent and comply with the study procedures.
  4. Nonpregnant, nonlactating, postmenopausal, surgically sterilized, female patients, or using a medically acceptable form of birth control, as determined by the investigator.
Exclusion
  1. Known conditions that would interfere with the evaluation of acne vulgaris. Such conditions include but are not limited to the following: rosacea; seborrheic dermatitis; perioral dermatitis; corticosteroid-induced acne or folliculitis; carcinoid syndrome; squamous cell carcinoma; mastocytosis; acneiform eruptions caused by make-up or medication; bacterial folliculitis; facial psoriasis; and facial eczema.
  2. Subjects allergic to herbal products or any component of the study product.
  3. Subjects who had been treated topical or oral corticosteroids within 14 days prior to baseline.
  4. History of uncontrolled disease or immune-deficient disorder.
  5. Any feature in the test areas (face) that according to the investigator, may influence the results, for example, but not limited to moles, tattoos, scars, irritated skin, scratches, cuts, and excess hair.
  6. Known HIV or Hepatitis B positive or any other immunocompromised state.
  7. Female subjects who are pregnant, nursing, or planning to become pregnant during study participation.
  8. Currently participating or having participated in another clinical trial during the last 3 months prior to the beginning of this study.
  9. Any additional condition(s) that in the investigator's opinion would warrant exclusion from the study or prevent the subject from completing the study.
